# Supplementary material for: Regulation of myoepithelial differentiation
Source: PLoS One. 2022 May 26;17(5):e0268668. doi: 10.1371/journal.pone.0268668 (PMC9135247; doi:10.1371/journal.pone.0268668)
Supplement: S1 Appendix — (PDF) [file pone.0268668.s001.pdf]

Table S1: Antibody Dilutions & Applications

Antibody	Company	Catalog #	Dilution	Application
**Primary Antibodies**				
αSMA	Sigma Aldrich	A5228	1:1000	ICC
TAZ	Sigma Aldrich	T4077	1:200	ICC
Calponin	Abcam	Ab46794	1:600	ICC
**Secondary Antibodies**				
AF568 Donkey anti-Rabbit	Alexa-Fluor	A10042	1:1000 (2D)<br>1:500 (3D)	ICC
AF488 Donkey anti-Mouse	Alexa-Fluor	A21202	1:1000 (2D)<br>1:500 (3D)	ICC
DRAQ5	Cell Signaling Technology	4084	1:1000 (2D)<br>1:500 (3D)	ICC

Table S2: qPCR Primer Sequences

Gene Product	Strand	Sequence (5'-3')
αSMA	Forward	GTCCCAGACATCAGGGAGTAA
	Reverse	TCGGATACTTCAGCGTCAGGA
AQP5	Forward	AGAAGGAGGTGTGTTCAGTTGC
	Reverse	GCCAGAGTAATGGCCGGAT
Calponin	Forward	GCACATTTTAACCGAGGTCCT
	Reverse	CTGATGGTCGTATTTCTGGGC
CTGF	Forward	CTCCACCCGAGTTACCAATG
	Reverse	TGGCGATTTTAGGTGTCC
CYR61	Forward	ACCAATGACAACCCAGAGTG
	Reverse	AAGTAAATCTGACTGGTTCTGGG
Integrin β4 subunit	Forward	ACTCCATGTCTGACGATCTGG
	Reverse	GGGACGCTGACTTTGTCCAC
TAZ	Forward	GTGTGCCCAATGCACTGA
	Reverse	TGACGCATCCTAATCCTCTCTC
α-actin	Forward	GGCTGTATTCCCCTCCATCG
	Reverse	CCAGTTGGTAACAATGCCATGT

Table S3: siRNA Sequences

Gene Name	Catalog Number	Company	Sequence
WWTR1 (TAZ), Sequence 1	Custom	Dharmacon	CAGAAUGACUUUAGAGAAUUU
WWTR1 (TAZ), Sequence 2	J-041057-09- 0002	Dharmacon	CAAUUUAUGUCCACGUUAA

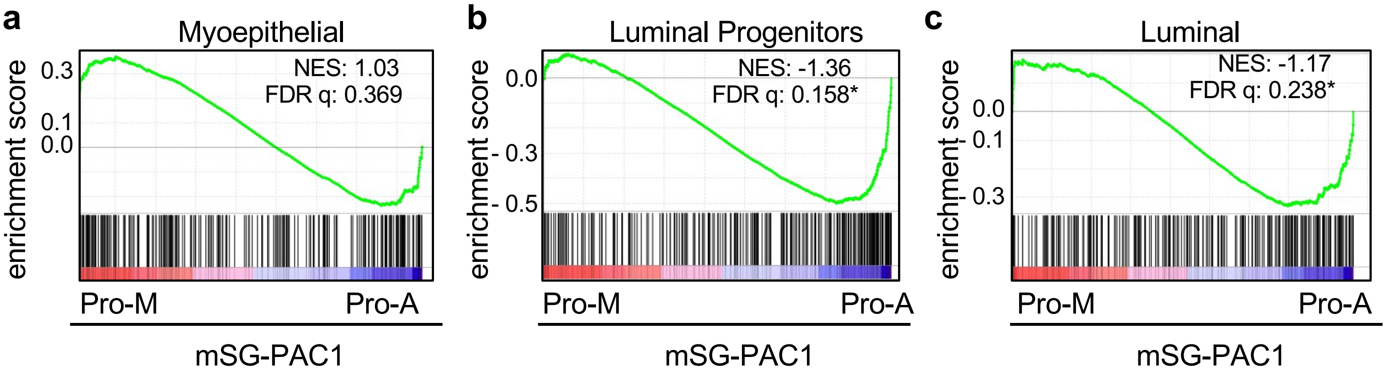

Supplementary Figure 1. Distinct differentiation programs are engaged in mSG-PAC1 spheroids cultured in Pro-A and Pro-M. GSEA was performed using gene sets derived from single cell RNA sequencing (scRNAseq) from murine mammary gland for myoepithelial cells and luminal cells from pregnant females and luminal progenitor cells from virgin females. Data sets were downloaded from Supplemental Table 5 data for each cell type[38]. The analysis was performed using the 250 genes with the lowest adjusted p-value. **(a)** Myoepithelial specific genes are modestly, but not significantly enriched in mSG-PAC1 spheroids cultured in Matrigel in Pro-M medium (NES = 1.03, FDR q-value = 0.369). **(b)** Genes specific to luminal progenitor (NES = -1.36, FDR q-value = 0.158) and **(c)** mature luminal cells (NES= -1.17, FDR q-value = 0.238) are significantly enriched mSG-PAC1 spheroids cultured in Matrigel in Pro-A medium (FDR q-value < 0.25 represent significant enrichment.

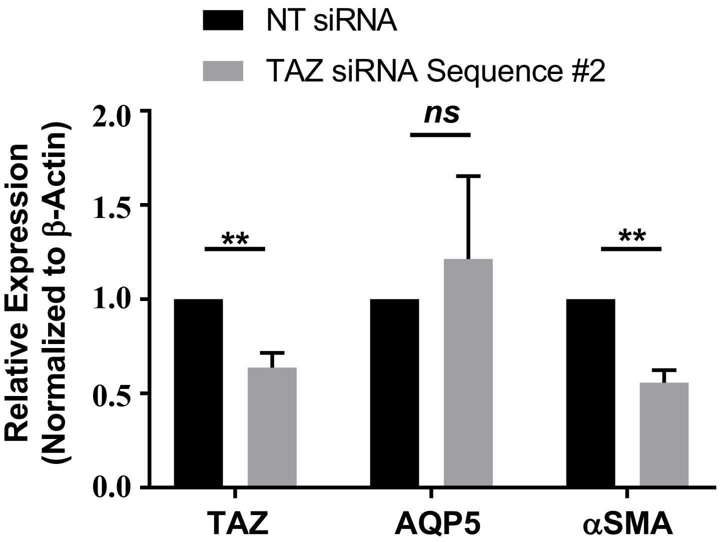

Supplementary Figure 2. TAZ depletion using a second siRNA sequence also inhibits the expression of αSMA without significantly affecting the expression of AQP5.

a

b

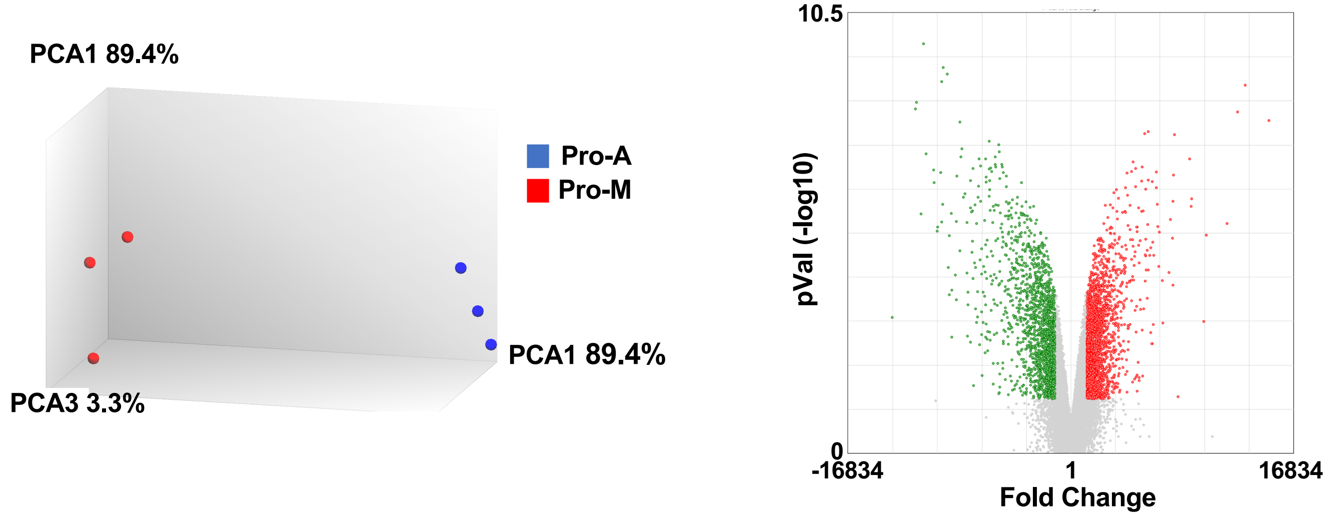

Supplementary Figure 3. (a) Principal component analysis (PCA) was performed to examine the variance between samples. **(b)** Volcano plot for differentially expressed genes in spheroids cultured in Pro-M compared to Pro-A medium. Genes increased by a factor of greater than 2-fold are shown in red and those that decreased by a factor of greater than 2-fold are shown in green.
