# Supplementary material for: Regulation of myoepithelial differentiation
Source: PLoS One. 2022 May 26;17(5):e0268668. doi: 10.1371/journal.pone.0268668 (PMC9135247; doi:10.1371/journal.pone.0268668)
Supplement: Attachment — Submitted filename: Response to Reviewers.docx [file pone.0268668.s002.docx]

The authors thank the reviewers for their helpful comments and suggestions. We addressed these below. Please note that because of the addition of new data, old Fig.5 became too large to upload. Data in old Fig. 5 together with new data are now presented in Figs. 5 and 6. Old Fig. 6 is now Fig. 7. Additionally, we edited the text to reflect changes in response to reviewers’ comments and also to correct typographical errors and to improve readability.

**REVIEWER 1**

**Comment 1**: Readers encounter the mSG-PAC1 cell line name on page 4…. Help your readers with a brief explanation and reference in the results.
**Response**: In our revised manuscript, we describe how we established mSG-PAC1 cells at the end of the Introduction together with the citation.

**Comment 2**: In the paper the changes in levels of aSMA, b4, aquaporin 5, calponin and YAP are through RNA analysis of the spheroids… Immunohistochemistry of these proteins on the spheroids should be supplied to show where the expression of these proteins is occurring. This is especially true for integrin beta4 which is an epithelial marker not a myoepithelial marker.

**Response**: This data has been provided in revised Figure 1, with the exceptions of the b4 subunit, which we did not detect at the protein level. The reviewer is correct that b4 is a marker for basal epithelial cells however it is also a marker for mature myoepithelial cells (see ref #36)

**Comment 3**:The data on TAZ transition to the nucleus, required for transcriptional activity, should be presented more clearly by showing images of just the TAZ staining.

**Response:** These images are provided in our new Figure 6. Old Figure 6 is now Fig. 7.

**Comment 4**: Where is the link to the uploaded transcriptional data for the other changed genes? Isn’t it possible that there are other genes that have changed that may also have implications for the myoepithelial phenotype?

**Response:** The link to our transcriptional data is provided in our revised manuscript. Yes, it very likely that other genes have changed that also have implications for the myoepithelial phenotype.

**REVIEWER 2**

**Comment 1:** Figure 1A (particularly the PRO-A condition) would benefit from addition of nuclear staining. It kind of looks like a hazy green blob, even though I am pretty sure it is an acinus.

Response: We have revised Figure 1 to address this and other issues.

**Comment 2:** Figure 1F would be substantially improved by showing every data point that was used to derive the mean w/h ratio. In its current form it is impossible to understand how the error bars are so tight, and why the statistic looks so strong. Perhaps it really is that good, but cells in nature tend towards variation in morphometrics.

**Response**: We have revised this graph.

**Comment 3**: There is no reference in the text to Figure 5C.

**Response**: Fig.5 has been separated into new Fig. 5 and new Fig. 6 Old Fig. 6 is now Fig. 7. All panels are now appropriately referenced in the text.

**Comment 4**: Statements about the subcellular localization of TAZ in developing glands in vivo (Figure 5) or in the mSG-PAC1 model (Figure 6) were not clearly supported by the microscopy. It was difficult to see supposed examples of localization at the cell-cell junction vs cytoplasm vs nucleus. e.g. Cytoplasmic vs Nuclear quantification would help, as would higher resolution images.

**Response**: We agree with the reviewer that we cannot justify the localization of TAZ at cell-cell junctions. We have removed this statement from our revised manuscript. We analyzed the nuclear localization of TAZ at E14, E15, E16, and E17. This analysis is provided in our new Fig. 6. Old Fig. 6 is now Fig 7 in our revised manuscript.
